# Dietary knowledge and practices among patients with diabetes in North Lebanon: the role of dietary counselling

**DOI:** 10.1017/jns.2025.10014

**Published:** 2025-06-06

**Authors:** Rosy Mitri, Zeina El-Ali

**Affiliations:** Department of Nutrition and Dietetics, Faculty of Health Sciences, Beirut Arab University, Tripoli, Lebanon

**Keywords:** Diabetes, Dietary counseling, Dietary knowledge, Dietary practices

## Abstract

The aim of the study is to assess the dietary knowledge and practices of Lebanese patients with diabetes not receiving dietary counselling in a low-income setting. A cross-sectional study was conducted among 317 Lebanese adult patients, not receiving dietary counselling in North Lebanon. Patients completed a questionnaire evaluating their sociodemographic, health and clinical characteristics as well as their dietary knowledge (DK) and practices. The mean total DK score as well as the mean scores for each category were calculated for the whole sample, transformed into percentages of maximal score and classified into poor (< 50%), good (50–75%) and adequate (> 75%). Patients had a good, but not optimal total DK (51.66%). Similarly, they also had a good knowledge related to carbohydrates (52.16%) and fat (52.5%), and to food type (60.83%). On the other hand, they had a poor knowledge about food choices (35.66%) and protein (44%). Linear regression analysis revealed that a higher educational level (β = 1.96, p < 0.001), choosing whole grains (β = 1.19, p = 0.002), living with a partner (β = 1.01, p = 0.007), being recently diagnosed with diabetes (β = –1.23, p = 0.012) were positively associated with a better DK. Furthermore, patients who suffered from type I diabetes had a better DK compared to those suffering from type 2 diabetes (β = –1.31, p = 0.016). The nutritional knowledge of the patients with diabetes not receiving dietary counselling is good but not optimal. Dietitians and doctors should collaborate to provide patient-centred and individualised dietary education to patients with diabetes.

## Introduction

Numerous Mediterranean countries have been witnessing a shift in dietary preferences in recent years from the traditional Mediterranean diet to the Westernized diet. This nutrition transition in diet and exercise patterns have resulted in the uprise of numerous health problems, which are the root cause of many metabolic disorders including diabetes.^(1)^ Concomitantly, among other regions, Middle Eastern and North African regions are of great concern as the prevalence of diabetes mellitus (DM) is projected to increase by 104% in 2040. As the matter of fact, this region is expected to have the second highest increase in the world.^(2)^

In Lebanon, according to a recent national survey, the prevalence of self-reported DM is estimated to be around 9.7%; and it is deemed that the actual total number of cases, including the undiagnosed ones, is even higher.^(3)^ Furthermore, most of the patients with diabetes are being followed up by endocrinologists, and only a minority is assessed by a primary healthcare physician.^(3)^ Bypassing the primary healthcare system can decrease the referral of the patients to other specialists who have important roles in diabetes care, including dietitians. Moreover, there is no medical coverage for nutrition and dietetic services. People seeking dietary counselling should pay the fees at their own expenses, which makes these services out of reach for a large section of the population. Due to all of the reasons mentioned above, most of the patients with diabetes end up not consulting with a dietitian. A study conducted in 2013 in Beirut, the capital of Lebanon, revealed that 38.25% of the studied patients with diabetes used dietary counselling services, and only 33% of them have done it more than once.^(4)^ It is expected that this number is even lower in other Lebanese regions due to economic and geographic factors.^(4)^ On the other hand, the effectiveness of appropriate medical nutrition therapy (MNT) in managing diabetes have been highlighted long time ago, and it has been concluded that MNT provided by an experienced registered dietician have an impact on diabetes management.^(5)^ Hence, due to lack of medical follow-up and dietary counselling, no wonder that 22% of patients reported having complications with retinopathy being by far, the most commonly reported one.^(3)^ Complications can create an additional economic burden at different levels especially in a country already undergoing an economic crisis like Lebanon.^(6)^

Patients who are not receiving dietary counselling may be unaware of the role of proper dietary management in the control of the diseases or may try to seek dietary information from non-formal sources like social media, friends or relatives. Therefore, we hypothesised that these patients have poor dietary knowledge and practices. To the best of our knowledge, there is no data about the dietary knowledge and practices of the patients with diabetes who are not receiving dietary counselling in North Lebanon. This geographical area is of specific importance in relation to the topic of interest. According to the latest World Bank report, 52% of North Lebanon residents are under the poverty line compared to just 2% in the capital Beirut.^(7)^ Thus, assessing the level of dietary knowledge (DK) among patients in this area is crucial to alert the appropriate authorities about the importance of designing awareness programmes regarding the role of nutrition in diabetes management. Moreover, evaluating the adequacy of their nutritional practices is of prime importance to examine the need for tertiary prevention programmes. Finally, determining the factors associated with poor knowledge and practices will facilitate the development of well-targeted policies to make dietary services accessible to those who need it. This is of special concern among individuals with limited financial means. Therefore, the aim of this study is to assess the degree of dietary knowledge and the appropriateness of dietary practices of patients with diabetes who are not receiving dietary counselling in a low-income setting and associated factors.

## Method

### Study design and participants

A cross-sectional study was conducted between February and May 2024 among 317 Lebanese patients suffering from diabetes, in North Lebanon. Patients with diabetes, aged 18 years and above, and attending primary health care centres (PHC) were interviewed by trained dietitians using a multi-component questionnaire. If a patient was followed by a dietitian, he was excluded.

### Sampling procedure

Sample size was calculated based on the prevalence of poor dietary knowledge among patients with diabetes in Saudi Arabia which was of 0.2857.^(8)^ A minimum number of 313 patients with diabetes was needed using the following formula: n = z^2^pq/d^2^ (n = sample size; z = 1.96 which corresponds to 95% confidence level; p = 28.57% corresponding to the prevalence of the primary outcome, which is dietary knowledge, taken from a previous study; q = 0.714 which corresponds to 1- p and d = 0.05 corresponding to the margin of error).^(8)^

A convenience sampling technique was used to recruit 317 patients with diabetes. These patients were recruited from three PHCs and one hospital located in North Lebanon. The directors of these healthcare centres were contacted in advance in order to obtain their consents. Patients were approached in the PHCs while waiting for their appointments for regular health check-ups at physicians’ offices. As for the hospital, the recruitment took place during a free blood test campaign designed for patients with diabetes. After explaining the aims of the study and the procedures, an informed consent was obtained from each study participant.

### Questionnaire

The measurement instrument was a multicomponent questionnaire that included four sections evaluating the sociodemographic characteristics, health and clinical characteristics, dietary practices, and dietary knowledge of the participants. The questionnaire was piloted in advance on a group of 20 patients with diabetes. Subsequently, few questions were modified in order to ensure their clarity. These included the following: (1) the question “*are you adopting any lifestyle modifications*?” was not clear, therefore, examples were provided “*diet and physical activity*”, (2) the question *“are you following any special diet?”* was further elaborated by adding the following examples “*low calorie diet; low-fat and carbohydrate controlled”,* and (3) the question “*how frequently do you consume alcohol?”* was removed because it was not culturally appropriate.

The questionnaire was administered to all study participants using a face-to-face-interview (Around 20 minutes for each participant).

#### Sociodemographic characteristics

These variables included age, gender (male/female), living arrangement (alone/with a partner), level of education (illiterate/primary/complementary/secondary/university). For the purpose of this analysis, the educational level categories were regrouped into 3 categories: low level (illiterate/primary/complementary), secondary and university. The crowding index, which is a tool used to assess the household socioeconomic status, was calculated by dividing the number of household residents by the total number of rooms (excluding kitchen and bathroom). The obtained score was categorised into low (≤ 2) and high (> 2).^(9)^

#### Health and clinical characteristics

These variables included type of diabetes (type I/type II/gestational diabetes), time since diagnosis (< 6 months/6 months–2 years/2–5 years/> 5 years), receiving treatment (yes/no), presence of other comorbidities (yes/no), smoking (active smoker/past smoker/non-smoker). The laboratory values pertaining to haemoglobin A1C (HbA1c), and lipid profile were extracted from the patient’s file and recorded as well. In order to classify patients as having poor metabolic control, the following cutoffs were used: ≥ 7% for HbA1c; ≥ 150 mg/dl for triglycerides; ≥ 200 mg/dl for total cholesterol and ≥ 100 mg/dl for Low density lipoprotein (LDL) cholesterol.^(10)^

#### Dietary practices

Patients were questioned whether they were following a special diet (yes/no), if they made some lifestyle modifications to control their disease (yes/no), and if they were practicing carbohydrate counting (never/sometimes/all the times). The latter question was categorised into yes (sometimes and all the time) and no (never). The patients were also asked to describe the frequency of choosing whole-grains over refined ones in their diet (never, rarely, often, always), as well as their sweets consumption (monthly or less, weekly, daily or more).

#### Dietary knowledge

A previously validated Dietary Knowledge questionnaire (DKQ) was used in this research.^(8)^ This tool comprised 21 multiple choice questions (MCQs) grouped into 5 categories assessing carbohydrates (6 items), lipids (4 items), proteins (2 items), food type (6 items) and food choices (3 items) knowledge. Each MCQ had only one correct answer. The responses were coded as 1 = correct, 0 = incorrect and/or I do not know. Each category score was calculated as the sum of all items of the corresponding category. The obtained scores ranged between 0–6 for carbohydrates and food type; 0–4 for lipids; 0–2 for proteins and 0–3 for food choices. The scores of the subcategories were summed up to obtain the total score that ranged from 0–21. The mean total score as well as the mean scores for each category were calculated for the whole sample. Subsequently, these means were transformed into percentages of maximal score. For example, if the mean was 4 over 6 for the carbohydrate knowledge category, the percentage of the mean score will be 66.6%. Higher score indicated a better nutritional knowledge. A score < 50% was classified as poor DK whereas, a score between 50–75% was considered as good DK and a scored > 75% represented adequate DK. The sources of nutritional knowledge were also investigated.

### Anthropometric measurements

Weight and height were measured for all participants. Each anthropometric measure was taken twice (using standardised techniques), and the average was recorded to the nearest 0.1 cm. Height was measured without shoes with the patient standing in the Frankfort horizontal plane. Body mass index (BMI) was then calculated using the following formula: weight(kg)/height(m^2^).^(11)^

### Statistical analysis

Data analysis was carried out using the Statistical Package for the Social Sciences (SPSS, version 23.0). Means (lowest–highest scores) were reported for continuous variables, while frequencies and percentages were used for categorical variables. The distribution of the knowledge score exhibited a bell-shaped curve, suggesting approximate normality. Given our large sample size, normality was assumed based on the Central Limit Theorem.^(12)^ To examine the association between DK and other categorical variables, we used independent samples t-tests for comparisons between two groups and one-way analysis of variance (ANOVA) for comparisons involving more than two groups. For age, Pearson correlation was used to assess the relationship with DK. Subsequently, a linear regression analysis was conducted to identify factors associated with DK. All predictors were first tested in simple linear regression, and only those with p < 0.05 in the bivariate analysis were included in the multiple regression model. The normality of residuals was assessed, and the F-statistic for the adjusted multiple regression model was 5.16 (p < 0.001). A p-value of less than 0.05 was considered statistically significant.

## Results

The general characteristics of the study population are presented in Table 1. The mean age of the patients was 59.29 (27–87) years. The sample included 128 males (40.4%) and 189 females (59.6%). The majority of the participants were living with a partner (80.1%). A low level of education (illiterate/primary/complementary) was reported by 68.5% of the patients with diabetes. Around 61.5% of the patients have been diagnosed with diabetes more than 5 years ago, with the majority of them (91.2%) suffering from other chronic diseases such as cardiovascular and kidney diseases. Overweight and obesity accounted for 82.1% of the sample. Additionally, 63.9% of them had a poor glycemic control. Poor dietary practices were noted among the majority of the patients with only 20.5% following a special diet, either a low-calorie diet, carbohydrate (CHO) controlled, or low in fat. The other health care professionals, like physician or pharmacist, were the number one source of dietary knowledge (68.5%). Only 13.9% of the patients counted carbohydrates, while around half of them (47%) never consumed whole grains.

**Table 1.** General characteristics of patients with diabetes (n=317)

Variables	n (%)
Age	
Mean [Lowest–Highest]	59.29 [27–87]
Gender	
Male	128 (40.4)
Female	189 (59.6)
Living arrangement	
Living alone (single/divorced)	63 (19.9)
Living with a partner	254 (80.1)
Educational level	
Low level (illiterate/primary/complementary)	217 (68.5)
Secondary	63 (19.9)
University	37 (11.7)
Crowding Index	
Low (≤ 2persons/room)	220 (69.4)
High (> 2 persons/room)	97 (30.6)
Type of diabetes	
Type I	27 (8.5)
Type II	287 (90.5)
Gestational diabetes	3 (0.9)
Time since diagnosis	
< 6 months	35 (11.0)
6 months–2 years	30 (9.5)
2–5 years	57 (18.0)
> 5 years	195 (61.5)
Presence of chronic diseases	
Yes	289 (91.2)
No	28 (8.8)
Smoking	
Active smoker	153 (48.3)
Past smoker	25 (7.9)
Non-smoker	139 (43.8)
BMI^a^	
Underweight	3 (0.9)
Normal weight	54 (17.0)
Overweight	101 (31.9)
Obese	159 (50.2)
Treatment (Medications/Insulin)	
Yes	298 (94.0)
No	19 (6.0)
Glycemic Control (HbA1c^b^)	
Poor controlled (≥ 7%)	191 (63.9)
Good control (< 7%)	
Triglycerides	
Good control	126 (43.9)
Poor control (≥ 150 mg/dl)	161 (56.1)
Total cholesterol	
Poor control (≥ 200 mg/dl)	91 (31.8)
LDL^c^	
Poor control (≥ 100 mg/dl)	141 (49.3)
Lifestyle modifications (diet & physical activity)	
Yes	103 (32.5)
No	214 (67.5)
Special diet	
Yes	65 (20.5)
No	252 (79.5)
Source of Nutrition Knowledge	
Other healthcare professionals	217 (68.5)
Non-health care professionals	100 (31.5)
CHO counting at each meal	
Yes (sometimes/ all the times)	44 (13.9)
No, never	273 (86.1)
Whole grains choices	
Never	149 (47.0)
Rarely	54 (17.0)
Often	43 (13.6)
Always	71 (22.4)
Sweets consumption	
Monthly or less (2–3times/month or less)	238 (75.1)
Weekly (2–3 times/week)	40 (12.6)
Daily or > 1 daily	39 (12.3)

a BMI: body mass index. BMI categorised as follows: < 18.5 kg/m^2^ Underweight; 18.5–24.9 kg/m^2^ Normal weight; 25–29.9 kg/m^2^ overweight; ≥ 30 kg/m^2^ obese.

b HbA1C: Glycated haemoglobin.

c LDL: Low density lipoprotein.

The mean DK score out of 21 questions was 10.85 (4–17). In terms of percentages, Lebanese patients with diabetes who were not visiting a dietitian had a good DK (51.66%).

The mean knowledge score of patients in the carbohydrates group was 3.13 (0–6), which translates into a good knowledge (52.16%). More than 50% of the patients answered correctly to the majority of the questions in this category, however, only 21.1% identified correctly the food highest in sugar, and only 24.3% knew what is the effect of eating too much sugar. The mean knowledge score in the lipids and fats group was 2.1 (0–4) which translates into a good knowledge (52.5%), however just 8.5% and 29% of the patients knew which food is rich in cholesterol and fat respectively. The mean knowledge score in the protein group was 0.88 (0–2) which indicates a poor knowledge (44%). Similarly, the mean knowledge score in the food type group was 3.65 (0–6) which indicates a good knowledge (60.83%), however around a quarter of the participants (24.0%) identified food with the highest glycemic index correctly. Finally, the mean knowledge score of food choice group was 1.07 (0–3), which shows a poor knowledge (35.66%), with just 11.4% of patients knew which type of food can be eaten eat freely. Percentages of correct answers for each sub-category are represented in Table 2.

**Table 2.** Dietary knowledge among the patients with diabetes (n = 317)

	Correct answers
	n (%)
Carbohydrate knowledge	
Which of the following is highest in carbohydrates?	225 (71)
What is the effect of unsweetened fruit juice on blood glucose?	201 (63.4)
Which of these foods has the highest amount of sugar in them?	67 (21.1)
Eating too much sugary food may cause?	77 (24.3)
Breads, cereals, rice, and pasta are high in?	267 (84.2)
Whole-grain foods like brown rice and whole wheat bread are better choices than white rice and white bread because whole-grains contains?	156 (49.2)
Lipids and fats knowledge	
Which of the following is highest in fat?	92 (29.0)
Which of these foods has highest amount of fat in them?	278 (87.7)
Food that contains fats and oils gives us a lot of?	271 (85.5)
Which of the following foods contains most cholesterol?	27 (8.5)
Protein knowledge	
Which of the following contains highest amount of proteins?	126 (39.7)
Which of the following foods is a complete source of protein?	156 (49.2)
Food type knowledge	
A well-balanced diet includes?	149 (47.0)
HbA1c test has some relationship with your diet?	229 (72.2)
Most of the excess sodium in our diet comes from?	139 (43.8)
Which of the following has the highest glycemic index?	76 (24.0)
Which of the following contains a vitamin that is needed for good vision?	301 (95.0)
Do you think that justified consumption of vitamin, mineral, carbohydrates, fat and protein have direct effect on outcomes of diabetes mellitus?	264 (83.3)
Food choices knowledge	
The diabetics’ diet is?	201 (63.4)
Which of the following is a food which you can eat freely?	36 (11.4)
Which should be used to treat low blood sugar?	104 (32.8)

Table 3 reports the findings of the bivariate analysis exploring the factors associated with DK. Results revealed that higher age, higher level of education, living with a partner, gestational diabetes, following a special diet and always consuming whole grains were associated with higher dietary knowledge (p = 0.004, p < 0.001, p = 0.030, p = 0.021, p = 0.004, p = 0.011, respectively).

**Table 3.** Factors associated with dietary knowledge-bivariate analysis (n=317)

Variables	DK^d^ Mean (±SD)	p-value
Age		
Mean (±SD)	59.29 (±10.17)	0.004
Gender		
Male	10.82 (±2.73)	0.844
Female	10.88 (±2.90)	
Living arrangement		
Living alone (single/divorced)	10.14 (±2.89)	0.030
Living with a partner	11.04 (±2.79)	
Educational level		
Low level (illiterate/primary/complementary)	10.40 (±2.88)	< 0.001
Secondary	11.35 (±2.52)	
University	12.70 (±2.05)	
Crowding Index		
Low (≤ 2persons/room)	10.96 (±2.83)	0.297
High (> 2 persons/room)	10.60 (±2.81)	
Type of diabetes		
Type I	12.04 (±2.95)	0.021
Type II	10.72 (±2.79)	
Gestational diabetes	13.33 (±3.21)	
Time since diagnosis		
< 6 months	11.80 (±2.74)	0.050
6 months–2 years	11.13 (±2.96)	
2–5 years	11.25 (±2.86)	
> 5 years	10.86 (±2.83)	
Presence of chronic diseases		
Yes	10.83 (±2.89)	0.486
No	11.14 (±2.17)	
Smoking		
Active smoker	10.81 (±2.85)	0.515
Past smoker	11.01 (±2.90)	
Non-smoker	10.32 (±2.25)	
BMI^a^		
Underweight	12.00 (±1.73)	0.879
Normal weight	10.74 (±3.27)	
Overweight	10.80 (±2.72)	
Obese	10.91 (±2.77)	
Treatment (Medications/Insulin)		
Yes	10.80 (±2.81)	0.164
No	11.84 (±3.08)	
Glycemic Control (HbA1c^b^)		
Poor controlled (≥ 7%)	10.72 (±2.75)	0.847
Good control	10.66 (±2.83)	
Triglycerides		
Good control	10.58 (±2.90)	0.756
Poor control (≥ 150 mg/dl)	10.68 (±2.70)	
Total cholesterol		
Poor control (≥ 200 mg/dl)	10.46 (±20.81)	0.148
Good dietary control (≤ 200 mg/dl)	10.97 (±2.68)	
LDL^c^		
Poor control (≥ 100 mg/dl)	10.77 (±2.81)	0.426
Good control	10.50 (±2.75)	
Lifestyle modifications (diet & physical activity)		
Yes	11.09 (±2.80)	0.315
No	10.75 (±2.84)	
Special diet		
Yes	11.78 (±2.83)	0.004
No	10.62 (±2.79)	
Source of Nutrition Knowledge		
Other healthcare professionals	10.70 (±2.80)	0.149
Non-health care professionals	11.20 (±2.87)	
CHO counting at each meal		
Yes (sometimes/ all the times)	11.57 (±2.57)	0.057
No, never	10.74 (±2.86)	
Whole grains choices		
Never	10.40 (±2.84)	0.011
Rarely	10.72 (±2.50)	
Often	11.19 (±2.92)	
Always	11.72 (±2.82)	
Sweets consumption		
Monthly or less (2–3 times/month or less)	10.73 (±2.73)	0.377
Weekly (2–3 times/week)	11.30 (±3.06)	
Daily or > 1 daily	11.18 (±3.16)	

a BMI: body mass index. BMI categorised as follows: < 18.5 kg/m^2^ Underweight; 18.5–24.9 kg/m^2^ Normal weight; 25–29.9 kg/m^2^ overweight; ≥ 30 kg/m^2^ obese.

b HbA1C: Glycated haemoglobin.

c LDL: Low density lipoprotein.

d DK: Dietary knowledge.

Table 4 presents the results of the crude and adjusted linear regression models examining the factors associated with DK score. For simplicity, only significant predictors are shown in Table 3, while the simple regression results for all predictors are provided in Supplementary Table 1. After adjusting for all significant predictors, age was no longer significantly associated with knowledge (β = –0.02, p = 0.244). Living with a partner remained significantly associated with a higher knowledge score (β = 1.01, p = 0.007). Education level continued to have a strong association, with university-educated participants showing the highest knowledge scores (β = 1.96, p < 0.001). Participants with Type II diabetes had lower knowledge scores compared to those with Type I (β = −1.31, p = 0.016). Similarly, those who had been diagnosed for more than 5 years have significantly lower knowledge scores (β = −1.23, p = 0.012). The effect of following a special diet was no longer statistically significant (β = –0.6, p = 0.118). A positive trend was observed between whole grain choices and DK, but only those who always chose whole grains had a significantly higher knowledge score than those who never did (β = 1.19, p = 0.002).

**Table 4.** Factors associated with DK score—crude and adjusted linear regression models (n=317)

	Crude model				Adjusted model			
	β	Sig.	95% CI		β	Sig.	95% CI	
Age	–0.05	**0.004**	–0.08	–0.01	–0.02	0.244	–0.05	0.01
Living arrangement								
Living alone (single/divorced)								
Living with a partner	0.89	**0.025**	0.11	1.67	1.01	**0.007**	0.27	1.74
Educational level								
Low level (illiterate/primary/complementary)								
Secondary	0.95	**0.016**	0.18	1.72	0.81	**0.033**	0.06	1.55
University	2.3	**< 0.001**	1.35	3.26	1.96	**< 0.001**	1	2.93
Type of diabetes								
Type I								
Type II	–1.32	**0.020**	–2.43	–0.21	–1.31	**0.016**	–2.38	–0.24
Gestational diabetes	1.3	0.448	–2.06	4.65	0.26	0.875	–2.98	3.5
Time since diagnosis								
< 6 months								
6 months–2 years	–0.67	0.341	–2.04	0.71	–0.65	0.326	–1.95	0.65
2–5 years	–0.55	0.359	–1.74	0.63	–0.49	0.390	–1.6	0.63
> 5 years	–1.27	**0.015**	–2.28	–0.25	–1.23	**0.012**	–2.19	–0.27
Special diet								
Yes								
No	–1.17	**0.003**	–1.93	–0.4	–0.6	0.118	–1.36	0.15
Whole grains choices								
Never								
Rarely	0.32	0.472	–0.55	1.19	0.06	0.892	–0.77	0.89
Often	0.78	0.106	–0.17	1.73	0.26	0.577	–0.65	1.17
Always	1.32	**0.001**	0.52	2.11	1.19	**0.002**	0.43	1.95

The outcome variable is DK, treated as a continuous variable.

Significant p-values (p < 0.05) are highlighted in bold.

In the crude model, only significant p-values are presented (full simple regression results for all predictors are provided in Supplementary Table 1).

The adjusted model includes all predictors that were significant at the crude level.

## Discussion

To the best of our knowledge, this is the first study to explore DK and practices among Lebanese patients with diabetes who are not followed-up by a dietitian. Our results revealed that these patients had a good total DK score (51.66%) and good DK scores in the carbohydrates, fat and food type subcategories (52.16%, 52.5%, 50% and 60.83% respectively). On the other hand, they had a poor knowledge about food choices (35.66%) and protein (44%). Although the total DK score and the DK score about carbohydrates in our sample was higher than the one reported among Saudi Arabian patients (28.57% and 33.3% respectively), yet, the knowledge level in our sample was not adequate (scores well below 75%).^(8)^ Moreover, there were many gaps in their understanding about key facts related to the management of the disease and prevention of its complications such as dietary sources of sugar, fat and cholesterol as well as glycemic index of foods (only 21.1%, 29% 8.5% and 24% of correct answers respectively). Conversely, a Nigerian study revealed that 55.8% and 62.9% of the patients named correctly the sources of cholesterol and fat respectively.^(13)^ This difference could be attributed to the fact that 74.6% of the Nigerian population received dietetic counselling. Furthermore, poor dietary practices were found among the majority of the patients in our sample, for example, only 13.9% of the patients counted carbohydrates. These results highlight the importance of the dietetic counselling not only in delivering the appropriate information but also in changing the dietary practices.

Appropriate dietary interventions were found to be effective ways to change dietary behaviours among patients with type II diabetes.^(14)^ Moreover, behaviour changes, not solely acquiring information, is the wanted final outcome that will halt the progression of the disease and prevent its complications. As a matter of fact, a previous study found an improvement in blood glucose and HbA1c levels in a group of patients receiving dietary counselling compared to a group not receiving the counselling.^(15)^ Therefore, no wonder that 63.9% of our sample had poor glycemic control although, 94% of them were treated with insulin or medications. Thus, while the healthcare professionals were the primary source of information for most of the patients, the DK was not adequate. Many gaps should be filled to provide optimal knowledge to these patients and to empower them to translate their knowledge into actions that could improve their glycemic control.

### Factors associated with nutritional knowledge

In the present study, a higher educational level was linked to a better nutritional knowledge. Previous studies showed a proportional increase in nutritional knowledge with the level of educational achievement. In a sample of Australian adults, the results of the multiple regression analysis revealed a positive correlation between nutritional knowledge and educational status.^(16)^ Similar results were also reported in Jordan.^(17)^

Contrary to our expectations, Lebanese patients recently diagnosed with diabetes had a better DK compared with those diagnosed since many years ago (p = 0.009). Our results contradict the literature, where higher dietary knowledge was correlated with a longer duration since diagnosis in Jordan,^(17)^ and in the United Arab Emirates.^(18)^ A systematic review recommended increasing the frequency of contacts to increase the effectiveness of dietary interventions among diabetic patients.^(14)^ Therefore, future follow-up studies should be conducted to better understand the changes in the levels of DK among patients with time taking into account the frequency of follow-up visits after diagnosis.

Choosing whole grain was associated with DK. Indeed, research indicates that meal selection is influenced by information-based resources, such as culinary competence and food knowledge.^(19)^ Among a group consisting of 803 women, aged 18–39 years, an increase in the dietary knowledge was associated with a higher fruits and vegetables consumption.^(20)^ When a greater knowledge would undoubtedly make the patient treatment easier, but it would not always imply better compliance overall. For example, undergraduate students who knew the components of a heathy meal, did not make the right choice of a healthy snack.^(21)^ Similarly, in our study, the rest of the dietary practices such as CHO counting, and sweet consumption did not reveal any association with DK. Therefore, it is imperative to allocate more resources to translate the knowledge into practices using appropriate nutrition interventions.

Living with someone can be positively correlated with a better nutritional knowledge. Indeed, social network is an important tool that may help individuals learn about the causes and preventative measures for certain diseases.^(22)^ For example, Belgium women residing with a partner had a higher general nutritional knowledge compared to other living arrangements.^(20)^ Strengthening social support has been previously recommended to increase the effectiveness of nutrition interventions among patients with type 2 diabetes.^(14)^ Our results are aligned with these findings. Furthermore, patients with type I diabetes had better DK compared to patients with type 2 diabetes. In type 1 diabetes, insulin regimen cannot lead to optimal blood glucose control unless it is accompanied by appropriate dietary measures.^(23)^ In fact, carbohydrate counting is required in order to estimate prandial doses of insulin.^(24)^ Therefore, it is anticipated that most type I diabetes patients receive, at least, basic nutrition recommendations from their treating physicians that have improved their DK level. This can be investigated in future comparative studies.

### Strengths and limitations

The current study is the first to assess dietary knowledge and practices among Lebanese patients with diabetes who were not receiving formal dietetic counselling and follow-up. However, many limitations should be noted. First, this study was conducted among patients from a low-socioeconomic status attending primary heath care centers, in a single region in Lebanon, therefore the results cannot be generalised to the entire Lebanese population. The cross-sectional design of the study is another limitation to report.

### Conclusion and recommendations

Lebanese patients with diabetes had an overall good but not optimal dietary knowledge. Our results highlighted the importance of dietary counselling not only to provide information, but also to translate knowledge into action and improve to glycemic control. Therefore, as a first step, empowering the dietitian to be the main and the sole provider of nutritional information is warranted through appropriate referral by the treating physician. Furthermore, patients from all socioeconomic levels should have access to dietary counselling. This can be achieved by mandatory inclusion of dietetic services in all PHCs. Additionally, national awareness campaigns, led by a multidisciplinary team of healthcare professionals are needed in order to raise awareness about the role of proper dietary choices in managing the disease and preventing its complications.

## Supporting information

Mitri and El-Ali supplementary material Mitri and El-Ali supplementary material

## Supplementary material

The supplementary material for this article can be found at https://doi.org/10.1017/jns.2025.14
