# Supplementary material for: Dietary knowledge and practices among patients with diabetes in North Lebanon: the role of dietary counselling
Source: J Nutr Sci. 2025 Jun 6;14:e39. doi: 10.1017/jns.2025.10014 (PMC12187482; doi:10.1017/jns.2025.10014)
Supplement: Mitri and El-Ali supplementary material [file S2048679025100141sup001.docx]

**Supplementary table 1: Factors associated with Dietary Knowledge among patients with diabetes – Simple linear regression analysis (n=317)**

|  | **betta** | **p-value** | **95% CI** | |
| --- | --- | --- | --- | --- |
| **Age** | -0.05 | **0.004** | -0.08 | -0.01 |
| **Gender** |  |  |  |  |
| Male |  |  |  |  |
| Female | 0.06 | 0.845 | -0.58 | 0.7 |
| **Living arrangement** |  |  |  |  |
| Living alone (single/divorced) |  |  |  |  |
| Living with a partner | 0.89 | **0.025** | 0.11 | 1.67 |
| **Educational level** |  |  |  |  |
| Low level (illiterate/primary/complementary) |  |  |  |  |
| Secondary | 0.95 | **0.016** | 0.18 | 1.72 |
| University | 2.3 | **<0.001** | 1.35 | 3.26 |
| **Crowding Index** |  |  |  |  |
| Low (≤ 2persons/room) |  |  |  |  |
| High (>2 persons/room) | -0.36 | 0.297 | -1.04 | 0.32 |
| **Type of diabetes** |  |  |  |  |
| Type I |  |  |  |  |
| Type II | -1.32 | **0.020** | -2.43 | -0.21 |
| Gestational diabetes | 1.3 | 0.448 | -2.06 | 4.65 |
| **Time since diagnosis** |  |  |  |  |
| <6 months |  |  |  |  |
| 6 months - 2 years | -0.67 | 0.341 | -2.04 | 0.71 |
| 2 - 5 years | -0.55 | 0.359 | -1.74 | 0.63 |
| > 5 years | -1.27 | **0.015** | -2.28 | -0.25 |
| **Presence of chronic diseases** |  |  |  |  |
| Yes |  |  |  |  |
| No | 0.31 | 0.578 | -0.79 | 1.42 |
| **Smoking** |  |  |  |  |
| Active smoker |  |  |  |  |
| Past smoker | -0.49 | 0.423 | -1.69 | 0.71 |
| Non-smoker | 0.2 | 0.554 | -0.46 | 0.85 |
| **BMI** |  |  |  |  |
| Underweight | 1.26 | 0.455 | -2.06 | 4.57 |
| Normal weight |  |  |  |  |
| Overweight | 0.06 | 0.898 | -0.88 | 1 |
| Obese | 0.17 | 0.702 | -0.71 | 1.05 |
| **Treatment (Medications/Insulin)** |  |  |  |  |
| Yes |  |  |  |  |
| No | 1.05 | 0.118 | -0.27 | 2.36 |
| **Glycemic Control (HbA1c)** |  |  |  |  |
| Good control |  |  |  |  |
| Poor controlled (≥7%) | 0.07 | 0.845 | -0.59 | 0.72 |
| **Triglycerides** |  |  |  |  |
| Good control |  |  |  |  |
| Poor control (≥150 mg/dl) | 0.1 | 0.751 | -0.54 | 0.75 |
| **Total cholesterol** |  |  |  |  |
| Good control |  |  |  |  |
| Poor control (≥200 mg/dl) | 0.51 | 0.143 | -0.17 | 1.2 |
| **LDL** |  |  |  |  |
| Good control |  |  |  |  |
| Poor control (≥100 mg/dl) | 0.26 | 0.420 | -0.38 | 0.9 |
| **Lifestyle modifications (diet & physical activity)** |  |  |  |  |
| Yes |  |  |  |  |
| No | -0.34 | 0.318 | -1.01 | 0.33 |
| **Special diet** |  |  |  |  |
| Yes |  |  |  |  |
| No | -1.17 | **0.003** | -1.93 | -0.4 |
| **Source of Nutrition Knowledge** |  |  |  |  |
| Other healthcare professionals |  |  |  |  |
| Non-health care professionals | 0.5 | 0.144 | -0.17 | 1.17 |
| **CHO counting at each meal** |  |  |  |  |
| Yes (sometimes/ all the times) |  |  |  |  |
| No, never | -0.82 | 0.073 | -1.73 | 0.08 |
| **Whole grains choices** |  |  |  |  |
| Never |  |  |  |  |
| Rarely | 0.32 | 0.472 | -0.55 | 1.19 |
| Often | 0.78 | 0.106 | -0.17 | 1.73 |
| Always | 1.32 | **0.001** | 0.52 | 2.11 |
| **Sweets consumption** |  |  |  |  |
| Monthly or less (2-3times/month or less) |  |  |  |  |
| Weekly (2-3 times/week) | 0.57 | 0.240 | -0.38 | 1.52 |
| Daily or >1 daily | 0.45 | 0.360 | -0.51 | 1.41 |
